# Threonyl-tRNA synthetase activates STAT3 by a nontranslational mechanism

**DOI:** 10.1016/j.jbc.2025.111032

**Published:** 2025-12-09

**Authors:** Pallob Barai, Reean Abdullah, Shruti V. Bendre, Erin Weisser, Maxine J. van der Donk, Kent L. Wong, Adriana Reyes-Ordoñez, Erik R. Nelson, Jie Chen

**Affiliations:** 1 Department of Cell and Developmental Biology, University of Illinois at Urbana-Champaign, Urbana, Illinois, USA; 2 Department of Molecular and Integrative Physiology, University of Illinois at Urbana-Champaign, Urbana, Illinois, USA; 3 Department of Chemistry, Northwestern University, Evanston, Illinois, USA; 4 Cancer Center at Illinois, University of Illinois at Urbana-Champaign, Urbana, Illinois, USA; 5 Beckman Institute for Advanced Science and Technology, University of Illinois at Urbana-Champaign, Urbana, Illinois, USA; 6 Carl R. Woese Institute for Genomic Biology- Anticancer Discovery from Pets to People, University of Illinois at Urbana-Champaign, Urbana, Illinois, USA; 7 Division of Nutritional Sciences, University of Illinois at Urbana-Champaign, Urbana, Illinois, USA

**Keywords:** aminoacyl tRNA synthetase, TARS, JAK, STAT, scaffold, lung cancer

## Abstract

Signal transducer and activator of transcription 3 (STAT3) is a major regulator of cell proliferation and survival, often found to be aberrantly activated in cancer. Here, we identify threonyl-tRNA synthetase 1 (TARS1) as an activator of STAT3. Elevated TARS1 expression in lung cancer correlates with poor patient survival. We find that overexpression of TARS1 supports non–small cell lung cancer cell proliferation *in vitro*, tumor formation of xenografts in mice, and hyperactivity of STAT3. Catalytically inactive TARS1 promotes STAT3 activation and cell proliferation, indicating that TARS1 functions in a nontranslational manner. Mechanistically, TARS1 physically associates with both STAT3 and Janus kinase (JAK), and TARS1 activation of STAT3 requires basal JAK activity. We propose a scaffold model in which TARS1 promotes proximity of STAT3 to JAK and subsequent phosphorylation of STAT3. This model is supported by the results of reconstitution experiments expressing recombinant TARS1, STAT3, and JAK1 in noncancer cells. Our study uncovers a novel mechanism of STAT3 dysregulation in cancer and provides a strong basis for therapeutic targeting of the noncanonical function of a housekeeping protein.

The signal transducer and activator of transcription (STAT) family of proteins are key transcription factors that regulate a wide array of cellular and developmental processes (1, 2, 3, 4). Originally identified as the transcription factors responsible for interferon-responsive gene expression, STATs are phosphorylated and activated by Janus kinases (JAKs), a family composed of JAK1, JAK2, JAK3, and TYK2 (3, 5, 6). Upon cytokine binding to its cognate receptor, receptor-associated JAK becomes activated and phosphorylates receptor, which in turn recruits STAT to be phosphorylated by JAK. Phosphorylated STAT forms a dimer, translocates into the nucleus, and activates gene expression (1, 3, 6). STATs can also be activated directly by some receptor tyrosine kinases, such as epidermal growth factor receptor (EGFR) (7) as well as by the nonreceptor tyrosine kinase Src (8).

STAT3 plays multifaceted roles in biological processes ranging from embryogenesis to immunity and homeostasis of various tissues (9, 10). Y705 phosphorylation activates STAT3, and S727 phosphorylation modulates its activity (11). Notably, STAT3 is recognized as an oncogene because of its ability to drive oncogenic transformation both *in vitro* and *in vivo* when hyperactivated (12). Hyperactivation of STAT3 is found in many types of cancers, including breast, cervical, colon, pancreatic, ovarian, and lung cancers, as well as cancers of hematologic origin (13, 14, 15). Interleukin 6 (IL-6) is one of the most studied cytokines that activate STAT3 *via* JAK1, JAK2, or TYK2 (16), leading to the expression of genes that support cancer cell survival, proliferation, metastasis, and angiogenesis (16, 17, 18). More than 50% of non–small cell lung cancer (NSCLC) tumors and cell lines have elevated or constitutively active STAT3 (19). Potential upstream activators of STAT3 in lung cancer include IL-6, which has been reported to be constitutively produced in some lung cancer cell lines and lung cancer patients (20, 21), and EGFR, which is often overexpressed or mutated in the kinase domain in NSCLC cells (19). While the canonical mechanisms of STAT3 activation by receptors and JAKs are well characterized, there is potential for alternative modes of STAT3 activation or context-specific regulatory mechanisms given the ever-expanding complexity of the JAK–STAT signaling system (3).

Aminoacyl-tRNA synthetases (AARSs) are housekeeping enzymes that hydrolyze ATP and charge tRNAs with cognate amino acids, essential for protein synthesis (22). Numerous nontranslational functions of AARSs beyond the realm of protein synthesis have been identified, including regulation of gene expression, cytokine signaling, cell growth, angiogenesis, and inflammation (23, 24, 25, 26). Threonyl tRNA synthetase 1 (TARS1), a class II cytoplasmic AARS, has been reported to have noncanonical functions (27). For instance, TARS1 can assemble a cap-dependent translation initiation complex (28). In an entirely different context, TARS1 negatively regulates skeletal muscle cell differentiation through inhibiting JNK signaling (29). In both cases, the N-terminal noncatalytic UNE-T domain is critical for the functions of TARS1 (28, 29). In addition, TARS1 is found to be secreted by human vascular endothelial cells upon vascular endothelial growth factor stimulation, and exogenous TARS1 can stimulate endothelial cell migration and angiogenesis, although a molecular mechanism is not known (30). Our current study uncovers a novel mechanism of STAT3 activation by TARS1, which may operate in cancer as well as noncancer cells.

## Results

### TARS1 promotes cell proliferation and tumor formation in a nontranslational mechanism

In perusing the Human Protein Atlas (https://www.proteinatlas.org/) (31), we noticed that TARS1 mRNA expression is elevated in several NSCLC cell lines. We also found that TARS1 overexpression inversely correlated with lung cancer patient overall survival (Kaplan–Meier plot: https://kmplot.com/analysis/) (32). Notably, although seven other AARSs displayed a similar correlation, the others did not (Fig. S1). This observation implies that the canonical protein synthesis role of AARS may not underlie the correlation between AARS overexpression and lung cancer patient outcome.

**Figure 1:**
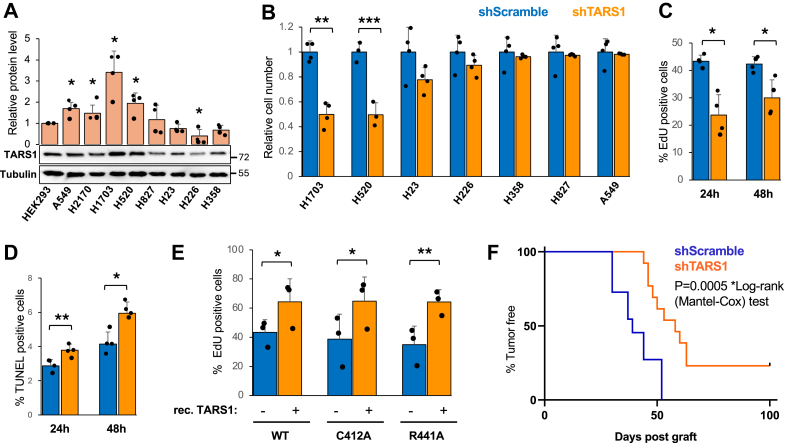
**TARS1 knockdown inhibits cell proliferation in a nontranslational manner.***A,* TARS1 protein levels were analyzed by Western blotting and quantified. Data were normalized against HEK293 (N = 4). *B,* cells were transduced with lentiviruses and selected in puromycin for 4 days. Cell viability data were normalized to control for each line (N = 4). *C* and *D,* H1703 cells were transduced with lentiviruses and selected in puromycin for 3 days, followed by EdU assay (*C*) or TUNEL assay (*D*) after 24 and 48 h (N = 4). *E,* H1703 cells transduced with shTARS1 lentivirus and selected in puromycin for 2 days were transfected with WT or mutant TARS1 for 3 days, followed by EdU assay (N = 4). *F,* H1703 cells transduced with lentiviruses and selected in puromycin for 4 days were grafted into mice. Tumor formation was monitored over time (N = 12 for each condition), with data presented in the Kaplan–Meier survival curve. Statistical analyses: one-sample *t* test for *A* and *B*; paired *t* test for C–E. ∗*p* < 0.05, ∗∗*p* < 0.01. EdU, 5-ethynyl-2'-deoxyuridine; HEK293, human embryonic kidney 293 cell line; TARS1, threonyl-tRNA synthetase 1.

To probe a potential function of overexpressed TARS1, we chose several NSCLC cell lines with diverse levels of TARS1 mRNA expression based on information in the Human Protein Atlas and examined TARS1 protein expression by Western blotting. As shown in Figure 1*A*, TARS1 protein levels were found to be elevated in A549, H2170, H1703, and H520 cells compared with human embryonic kidney 293 (HEK293) cells and significantly lower in H226 cells. To examine a correlation between TARS1 expression and cell proliferation, we knocked down TARS1 using a previously published lentivirus-delivered shRNA (29). A significant reduction in cell number was found upon TARS1 knockdown in H1703 and H520 cells (Fig. 1*B*). TARS1 protein levels were reduced to ∼10% to 40% by the shRNA, and it is important to note that the degree of TARS1 knockdown was not higher in H1703 and H520 than in the other cell lines (Fig. S2*A*). To assess whether the reduction in cell number was due to inhibited cell proliferation or increased cell death, we performed 5-ethynyl-2′-deoxyuridine (EdU) labeling assay and TUNEL assay in H1703 cells 24 and 48 h after TARS1 was sufficiently knocked down (3 days of puromycin selection following lentivirus transduction; Fig. S2*B*). The numbers of proliferating and apoptotic cells were significantly reduced (Fig. 1*C*) and increased (Fig. 1*D*), respectively. Since shTARS1 reduced proliferating cells from ∼40% to ∼20% and increased apoptotic cells only by 1% to 2%, the effect of TARS1 knockdown on overall cell numbers was primarily because of a reduction in cell proliferation.

The cell phenotype of TARS1 knockdown could have resulted from compromised global protein synthesis. To investigate that possibility, we made use of two mutants of TARS1 (C412A and R441A) that were expected to be catalytically inactive (29, 33). Aminoacylation assays with immunoprecipitated recombinant proteins confirmed that the R441A mutant had drastically reduced activity, whereas the C412A mutant activity was variable and not significantly different from the WT (Fig. S3). In TARS1 knockdown H1703 cells, expression of WT recombinant TARS1 (mouse complementary DNA, resistant to the shRNA) promoted proliferation as revealed by EdU assays, and each of the TARS1 mutants also promoted cell proliferation to the same extent as WT (Fig. 1*E*). At least, the result of R441A suggests that TARS1 has a role in promoting cancer cell proliferation through a nontranslational mechanism.

To probe the role of TARS1 in promoting cancer cell growth *in vivo*, we performed mouse xenograft experiments by grafting nude mice with H1703 cells treated with shTARS1 or shScramble lentivirus. We found that mice that were injected with TARS1 knockdown cells took longer to form palpable tumors than mice injected with control cells (Fig. 1*F*). Furthermore, while all the mice injected with control H1703 cells developed tumors, 23% of the mice injected with TARS1 knockdown cells did not form any tumor within 100 days of grafting (Fig. 1*F*). We observed no difference in histological features of the shScramble and shTARS1 tumors (Fig. S4). These observations indicate that tumor formation from the NSCLC cell line H1703 is dependent on elevated levels of TARS1.

### TARS1 promotes STAT3 activation

**Figure 2:**
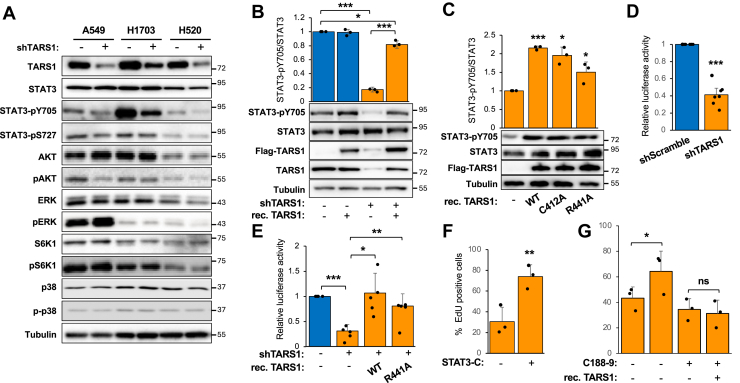
**TARS1 knockdown inhibits STAT3 activity.***A,* cells were transduced with shScramble (−) or shTARS1 (+) lentivirus and selected in puromycin for 4 days, followed by Western blot analysis. N = 3. Quantification is shown in Figure S5. *B,* H1703 cells were transduced with lentiviruses as in *A* and selected in puromycin for 2 days, followed by transfection of TARS1 for 3 days and Western blot analysis. Data were normalized to control (N = 3). *C,* H1703 cells transduced with shTARS1 lentivirus were treated as in *B*, followed by Western blot analysis. Data were normalized to control (N = 3). *D,* H1703 cells were transduced with lentiviruses, selected in puromycin for 2 days, and transfected with a STAT3 reporter for 2 days, followed by luciferase assays (N = 7). *E,* H1703 cells were treated as in *D*, but the STAT3 reporter was cotransfected with TARS1. Luciferase assay was performed (N = 5). *F,* H1703 cells were treated as in *C* and transfected with STAT3-C for 3 days, followed by EdU assay (N = 4). *G,* H1703 cells were treated as in *B*, with 2.5 μM C188-9 for the last 24 h, followed by EdU assay (N = 3). Data without inhibitor were the same as in Figure 1*E*. Statistical analyses: two-way ANOVA followed by Tukey’s post hoc test for *B* and *E*; one-sample *t* test for *C* and *D*; paired *t* test for *F* and *G*. ∗*p* < 0.05, ∗∗*p* < 0.01, and ∗∗∗*p* < 0.001. EdU, 5-ethynyl-2'-deoxyuridine; STAT3, signal transducer and activator of transcription 3; TARS1, threonyl-tRNA synthetase 1.

To decipher the molecular mechanism by which TARS1 regulates cell proliferation nontranslationally, we set out to study the effect of TARS1 knockdown on signaling pathways commonly known to control cell proliferation. We performed experiments in the two NSCLC cell lines in which TARS1 knockdown inhibited cell growth—H1703 and H520 (Fig. 1*B*). A549 cells, which were not affected by the knockdown, were also included for comparison. The activity levels of the following signaling proteins were examined by Western blotting for their phosphorylation: ERK, p38, AKT, mTORC1 (S6K1), and STAT3. Previously, we had reported TARS1 regulation of JNK signaling in myoblasts (29), but phospho-JNK was not detected reliably in these NSCLC cells. The only consistent change observed in both H1703 and H520 (but not A549) cells was with STAT3; Y705 phosphorylation, which is critical for STAT3 dimerization and activation, was significantly diminished by TARS1 knockdown (Figs. 2*A* and S5). S727 phosphorylation of STAT3 was not affected.

As expected, expression of recombinant WT-TARS1 restored the level of pY705-STAT3 in H1703 cells with endogenous TARS1 knocked down (Fig. 2*B*). Importantly, the catalytically inactive mutants of TARS1, C412A, and R441A also activated pY705-STAT3 (Fig. 2*C*), consistent with the notion that the effect of TARS1 on STAT3 is through a nontranslational mechanism. Next, we measured transcriptional activity of STAT3 using a luciferase reporter (34). Our results showed that TARS1 knockdown drastically reduced STAT3 reporter activity in H1703 cells (Fig. 2*D*) and that expression of WT-TARS1 or catalytically inactive mutant TARS1 (R441A) rescued the activity (Fig. 2*E*). Finally, to ascertain the role of STAT3 in mediating TARS1 regulation of cell proliferation, we expressed the constitutively active STAT3-C (12) in TARS1 knockdown H1703 cells. Indeed, we observed that STAT3-C enhanced proliferation of TARS1-knockdown cells (Fig. 2*F*). On the other hand, in the presence of the STAT3 inhibitor C188-9, TARS1 overexpression no longer promoted proliferation (Fig. 2*G*). Taken together, our observations strongly suggest that TARS1 activates STAT3 in a nontranslational manner to promote cell proliferation.

### TARS1 interacts with STAT3

**Figure 3:**
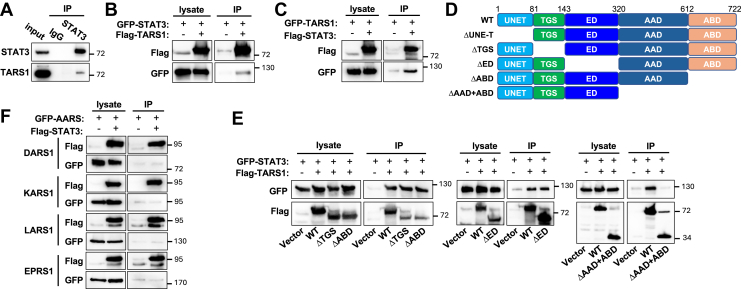
**TARS1 physically interacts with STAT3.***A,* H1703 cells were subjected to immunoprecipitation (IP) with anti-STAT3 followed by Western blotting. N = 2. *B*, *C*, *E*, *F*, HEK293 cells were transfected for 2 days with various constructs as indicated, followed by anti-FLAG IP and Western blotting. N = 3. *D,* TARS1 domains and the truncation constructs used in this study. DARS1, aspartyl-tRNA synthetase 1; EPRS1, glutamyl-prolyl-tRNA synthetase 1; HEK293, human embryonic kidney 293 cell line; KARS1, lysyl-tRNA synthetase 1; LARS1, leucyl-tRNA synthetase 1; STAT3, signal transducer and activator of transcription 3; TARS1, threonyl-tRNA synthetase 1.

To elucidate the mechanism by which TARS1 regulates STAT3, we tested the possibility of physical interaction between the two proteins. We found that endogenous TARS1 and STAT3 interacted in coimmunoprecipitation (co-IP) experiments performed with H1703 cell lysates (Fig. 3*A*). This interaction was further confirmed by co-IP of recombinant proteins expressed in HEK293 cells: IP of FLAG-TARS1 brought down GFP-STAT3 (Fig. 3*B*) and IP of FLAG-STAT3 brought down GFP-TARS1 (Fig. 3*C*). We went on to map the domain of TARS1 responsible for the interaction by testing several truncation constructs (Fig. 3*D*). As shown in Figure 3*E*, deletion of the TGS (ThrRS, GTPase, and SpoT) domain, editing domain (ED), or anticodon-binding domain (ABD) did not affect TARS1 interaction with STAT3. On the other hand, a C-terminal truncation, which removed the aminoacylation domain (AAD) and ABD, abolished interaction with STAT3, suggesting that AAD is necessary for TARS1 interaction with STAT3. We were not able to test whether AAD is sufficient for interaction with STAT3 because AAD alone did not express well in cells.

Given our observation that the interaction with STAT3 involved TARS1’s catalytic core domain, which is conserved among class II AARSs, we asked whether other AARSs may also interact with STAT3. To that end, we tested recombinant aspartyl-tRNA synthetase 1 (class II), lysyl-tRNA synthetase 1 (class II), leucyl-tRNA synthetase 1 (class I), and glutamyl-prolyl-tRNA synthetase 1 (dual enzyme) in co-IP experiments with recombinant STAT3. As shown in Figure 3*F*, none of those AARSs was pulled down by FLAG-STAT3. Therefore, interaction with STAT3 appears to be a feature unique to TARS1 among the AARSs.

### TARS1 acts as a scaffold to activate STAT3 through JAKs

**Figure 4:**
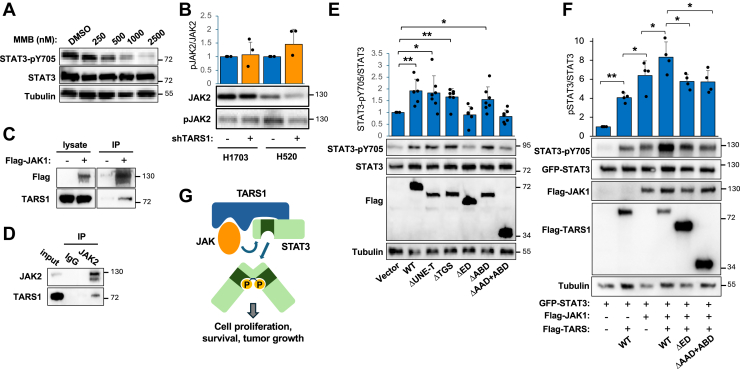
**TARS1 act as a scaffold to activate STAT3 by JAKs.***A,* H1703 cells were treated with momelotinib (MMB) at concentrations indicated or dimethyl sulfoxide (DMSO) for 30 min, followed by Western blotting. N = 3. *B,* H1703 and H520 cells were transduced with shScramble (−) or shTARS1 (+) lentivirus and selected in puromycin for 4 days, followed by Western blotting and quantification (N = 3). *C,* HEK293 cells were transfected with FLAG-JAK1 (or empty vector) for 2 days, followed by anti-FLAG immunoprecipitation (IP). Endogenous TARS1 was detected by Western blotting. N = 3. *D,* H1703 cells were subjected to IP with anti-JAK2 followed by Western blotting. N = 2. *E,* H1703 cells were transduced with shTARS1 lentivirus and selected in puromycin for 2 days, followed by transfection with various TARS1 constructs for 3 days. Western blots were quantified, and the data were normalized against vector control (N = 7). *F,* HEK293 cells were cotransfected with various combinations of GFP-STAT3, FLAG-JAK1, and FLAG-TARS1 (WT or mutants) for 2 days, followed by Western blotting. Quantification data were normalized against GFP-STAT3 transfection alone (N = 4). *G,* a proposed model of TARS1 serving as a scaffold to activate STAT3. Statistical analyses: one-sample *t* test for *B* and *E*; paired *t* test for *F*. ∗*p* < 0.05, ∗∗*p* < 0.01. HEK293, human embryonic kidney 293 cell line; JAK, Janus kinase; STAT3, signal transducer and activator of transcription 3; TARS1, threonyl-tRNA synthetase 1.

Next, we asked which kinase might be responsible for STAT3 phosphorylation promoted by TARS1 in NSCLC cells. JAK1 and JAK2 have been reported to be upstream of STAT3 in various NSCLC cells (35, 36, 37). Indeed, the pan-JAK inhibitor momelotinib (38) inhibited pY705 of STAT3 in H1703 cells in a dose-dependent manner (Fig. 4*A*). However, TARS1 knockdown did not affect the activity of JAK2 in H1703 or H520 cells (Fig. 4*B*). We were not able to detect JAK1 activity, which could be due to a low level of pJAK1 in these cells and/or low avidity of the antibody used. IL-6 is known to activate STAT3 in many NSCLC cells, but IL-6 treatment did not markedly increase the level of pY705-STAT3 in H1703 cells (Fig. S6*A*), most likely because STAT3 was already hyperactivated in these cells. This observation is consistent with the reported IL-6 independence of H1703 cells (35). Interestingly, IL-6 fully protected pSTAT3 from TARS1 knockdown (Fig. S6*A*), suggesting that IL-6 is capable of activating STAT3 in H1703 and that this activation is independent of TARS1. Furthermore, we found that serum starvation did not affect pSTAT3 status in H1703 cells (Fig. S6*B*), ruling out any stimulatory factor in the serum to be responsible for the hyperactivity of STAT3. Taken together, our observations suggest that TARS1 activation of STAT3 is most likely dependent on the constitutive activity of JAK and independent of cytokine receptor signaling.

A simple model that could explain all our observations thus far would be that, when overexpressed such as in certain NSCLC cells, TARS1 serves as a scaffold to bring JAK and STAT3 into proximity, thus promoting STAT3 phosphorylation and activation by JAK. Consistent with this model, a recombinant JAK1 pulled down endogenous TARS1 in a co-IP assay (Fig. 4*C*). Importantly, IP of endogenous TARS1 also brought down endogenous JAK2 from H1703 cells (Fig. 4*D*). To test the scaffold model functionally, we examined the capacity of various TARS1 truncations to activate STAT3 by expressing the recombinant proteins in TARS1-knockdown H1703 cells, similar to the experiments described earlier (Fig. 2, *B* and *C*). As shown in Figure 4*E*, the UNE-T, TGS, and ABD domains were dispensable for TARS1’s effect on STAT3, whereas constructs lacking either ED or AAD no longer activated STAT3. Since AAD but not ED was involved in interacting with STAT3 (Fig. 3*E*), the requirement of ED for STAT3 activation suggests that ED may interact with JAK.

To further test our proposed model, we carried out a reconstitution experiment in HEK293 cells by expressing GFP-STAT3, FLAG-TARS1, and FLAG-JAK1. Expression of recombinant TARS1 enhanced phosphorylation of GFP-STAT3 (Figs. 4*F* and S7). Importantly, the JAK inhibitor (momelotinib) abolished TARS1-induced elevation of pSTAT3 (Fig. S7), suggesting that TARS1 activation of STAT3 is dependent on JAK. Overexpression of TARS1 also boosted recombinant JAK1 activation of pSTAT3, but the TARS1 mutants missing ED or AAD + ABD domains did not have any effect (Fig. 4*F*). Taken together, these observations are in full agreement with the scaffold model by which TARS1 promotes JAK activation of STAT3 (Fig. 4*G*).

## Discussion

Our study unveils a novel mechanism of STAT3 activation by TARS1. In some NSCLC cells where TARS1 expression is elevated, cell proliferation is dependent on the overexpressed TARS1. TARS1 knockdown in H1703 cells drastically reduces tumor formation and growth in a xenograft mouse model, consistent with poor patient survival in lung cancer with high TARS1 expression. Our biochemical analyses have led us to propose that the activation of STAT3 by TARS1 is dependent on basal activity of JAK, and that TARS1, acting in a nontranslational manner, serves as a molecular scaffold to enhance JAK phosphorylation of STAT3. Constitutively active or hyperactive STAT3 is found in many lung cancers. An important question is why not all the TARS1-overexpressing NSCLC cell lines we studied are dependent on TARS1 for STAT3 activity and cell proliferation. Both H1703 and H520 are derived from squamous cell carcinoma, which is a less common type of NSCLC than adenocarcinoma. Common drivers in lung adenocarcinoma (but not lung squamous cell carcinoma) include EGFR and K-Ras mutations, which can serve as the activating signal upstream of STAT3. The H1703 and H520 cells, in which TARS1 is found responsible for STAT3 constitutive activity, do not harbor either EGFR or K-Ras mutation (DepMap (39)), and they do not express notable levels of IL-6 (The Human Protein Atlas (31)) for autocrine signaling. Hence, it is tempting to speculate that overexpressed TARS1 may provide the alternative means to activate STAT3 in NSCLC cells without EGFR and K-Ras mutations. Future investigation taking advantage of resources such as the Cancer Dependency Map (39) and a recently developed deep learning model (40) can lead to a broader understanding of TARS1’s role in cancer.

Nontranslational functions of AARSs are often facilitated by appended domains outside the catalytic core of these enzymes (24, 41). Previously, we reported that TARS1 regulates JNK signaling through its appended domains (UNE-T and TGS) (29). However, in the current study, we find that those domains of TARS1 are dispensable for the activation of STAT3. Instead, the catalytic ED and AAD of TARS1 are necessary to exert the scaffolding function. The AAD (but not ED) is structurally conserved among class II AARSs (42), but we did not find STAT3 to interact with other AARSs tested (Fig. 3*F*). Therefore, it is conceivable that activation of STAT3 *via* a scaffolding mechanism is a unique function of TARS1, not shared by other AARSs. Several other AARSs also exhibit a strong correlation between their expression levels and lung cancer mortality (Fig. S1), and the underlying mechanisms will warrant future investigation. Targeting nontranslational functions of these house-keeping enzymes could offer novel anticancer strategies.

Data from our reconstitution experiments expressing recombinant TARS1, STAT3, and JAK in HEK293 cells also suggest that the scaffolding mechanism does not require a cancer-specific cellular context. It is likely that activation of STAT3 can occur under physiological conditions where the optimal concentrations of TARS1–STAT3–JAK are present to favor the scaffolding complex assembly. Identification of the types of cells, tissues, and physiological contexts in which TARS1 activates STAT3 can potentially lead to new biological insights. Although our current model does not involve receptor signaling, it is noted that JAK is often constitutively associated with cytokine receptors, raising intriguing questions that can guide future mechanistic dissections. For instance, what is the subcellular location where TARS1 functions as a scaffold? Are cytokine receptors involved in a ligand-independent manner?

## Experimental procedures

### Antibodies and plasmids

All antibodies used in this study were obtained from commercial sources (see supporting information for details). The sources or creation of plasmids are detailed in supporting information.

### Cell culture

Cells were maintained in Ham’s F-12K medium (for A549), RPMI1640 medium (for all other NSCLC cell lines), or Dulbecco's modified Eagle's medium (for HEK293), containing 4.5 g/l glucose, 10% fetal bovine serum, and 1% penicillin–streptomycin. All cells were cultured at 37 °C with 5% CO_2_. Transfection of H1703 and HEK293 cells was performed using Lipofectamine 3000 and polyethylenimine, respectively. See supporting information for details.

### Lentivirus-delivered RNA interference

shRNAs in the pLKO.1-puro vector were purchased from Sigma–Aldrich (MISSION The RNAi Consortium). shRNA details, lentiviral packaging, and viral transduction are described in supporting information.

### Cell viability assay, cell proliferation assay, TUNEL assay, luciferase assay, and immunofluorescence staining

See supporting information for details.

### Mouse xenograft

All protocols involving animals were approved by the Institutional Animal Care and Use Committee at the University of Illinois at Urbana-Champaign. See supporting information for details of the procedure performed.

### Cell lysis, IP, and Western blotting

See supporting information for details.

### Statistical analysis

All quantitative data were presented as mean ± SD. Statistical significance of the data comparison was analyzed by performing two-tailed paired, unpaired, or one-sample *t* test, or two-way ANOVA followed by Tukey’s post hoc test for multiple comparisons. Differences between groups were considered significant when *p* < 0.05. The analyses were performed using Excel (Microsoft) and GraphPad Prism 8.0 (GraphPad Software, Inc).

## Data availability

All supporting information are included in the main article and its supporting information.

## Supporting information

This article contains supporting information (12, 29, 43, 44, 45, 46, 47, 48, 49, 50).

## Conflict of interest

The authors declare that they have no conflicts of interest with the contents of this article.
