# Supplementary material for: Threonyl-tRNA synthetase activates STAT3 by a nontranslational mechanism[image]
Source: J Biol Chem. 2025 Dec 9;302(2):111032. doi: 10.1016/j.jbc.2025.111032 (PMC12818216; doi:10.1016/j.jbc.2025.111032)
Supplement: Supplemental Figures [file mmc1.pdf]

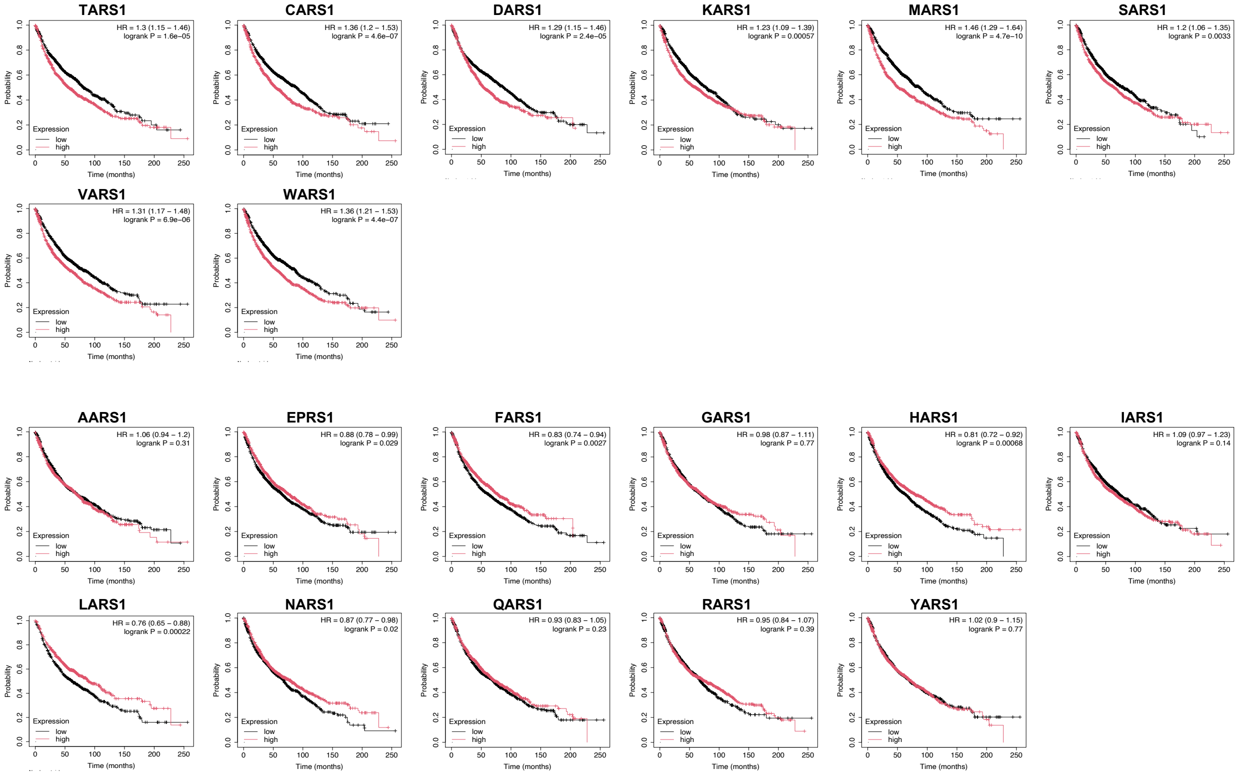


**Figure S1: AARS expression and patient survival in lung cancer**

KM-plots for all AARSs in lung cancer are shown, separated into two groups – high expression correlates with poor patient overall survival (upper group) or not (lower group).

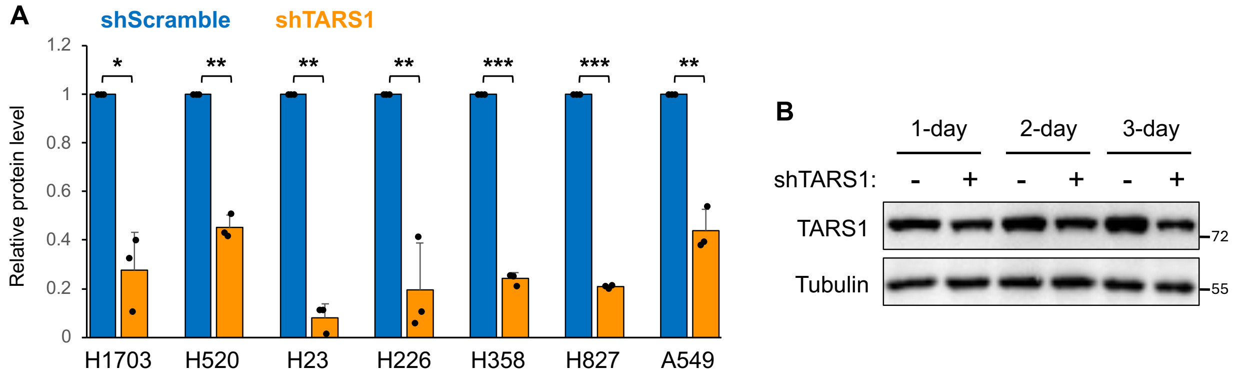


**Figure S2: TARS1 knockdown efficiency**

**(A)** Various cell lines were transduced by lentivirus shScramble or shTARS1 overnight, selected in puromycin for 4 days, and then subjected to western blot analysis followed by densitometry quantification. Data were normalized to shScramble for each cell line. N = 3. One-sample t-test was performed. *$p < 0.05$, **$p < 0.01$, ***$p<0.001$.

**(B)** H1703 cells were transduced by lentivirus shScramble or shTARS1 overnight, and selected in puromycin for 1, 2, or 3 days, followed by western blot analysis. Results shown are representative of 3 independent experiments.

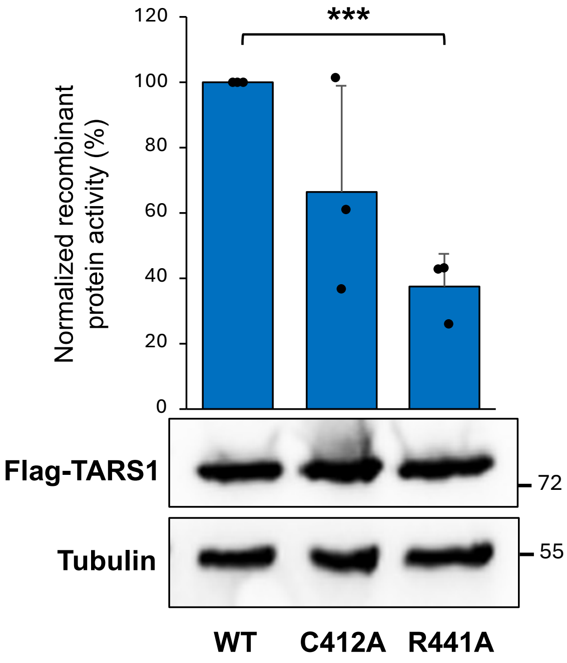


**Figure S3: Aminoacylation assay**
HEK293 cells were transfected with Flag-tagged WT or mutant TARS1 for 24 hours, followed by cell lysis and anti-Flag IP. The immunocomplexes were subjected to tRNA aminoacylation assay, and activities were normalized to WT as 100%. N = 3. One-sample t-test: ****p* < 0.001.

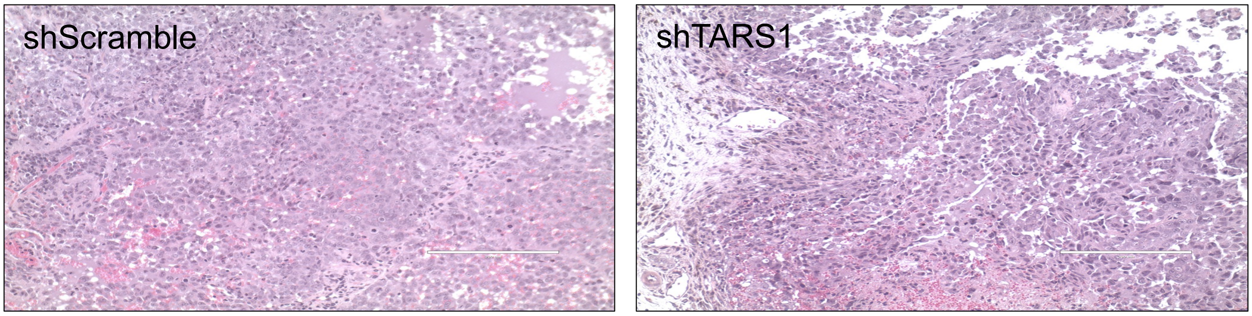


**Figure S4: Histology of xenographed tumors**
H&E images of representative tumors from Fig. 1F are shown. Scale bars: 200 µm.

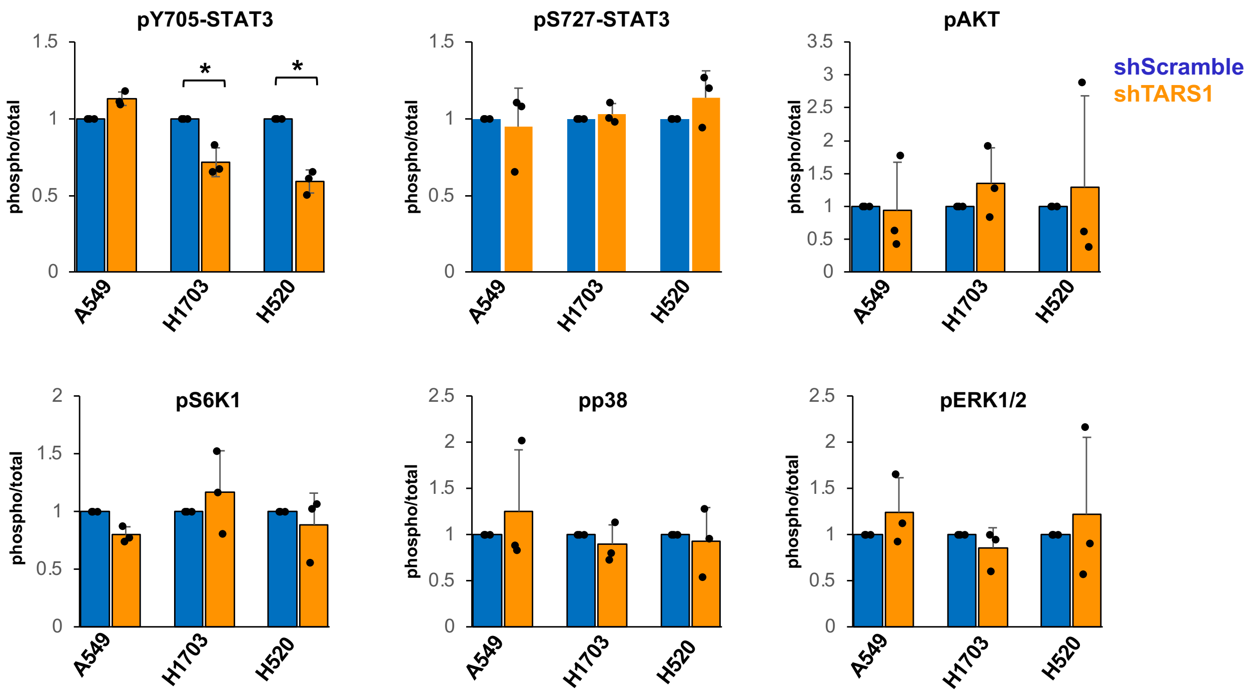


**Figure S5: TARS1 knockdown effects on signaling proteins**
Densitometric quantification of western blots shown in Fig. 2A. Data were normalized to shScramble for each cell line. N = 3. One sample t-test was performed. *$p$ < 0.05.

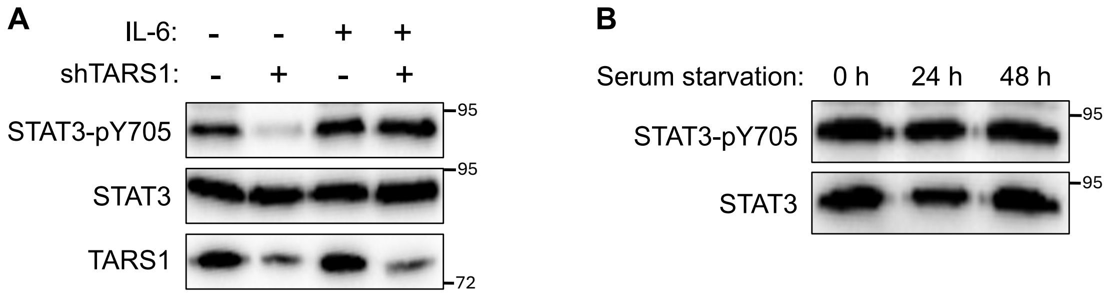


**Figure S6: TARS1 activation of STAT3 in H1703 cells is independent of upstream signals**
**(A)** H1703 cells were transduced by lentivirus shScramble (-) or shTARS1 (+) overnight, selected in puromycin for 4 days, and then stimulated with 10 nM IL-6 for 30 min followed by western analysis. Results shown are representative of 3 independent experiments.
**(B)** H1703 cells were cultured in serum-free medium for 24 or 48 hours, followed by western analysis. Results shown are representative of 3 independent experiments.

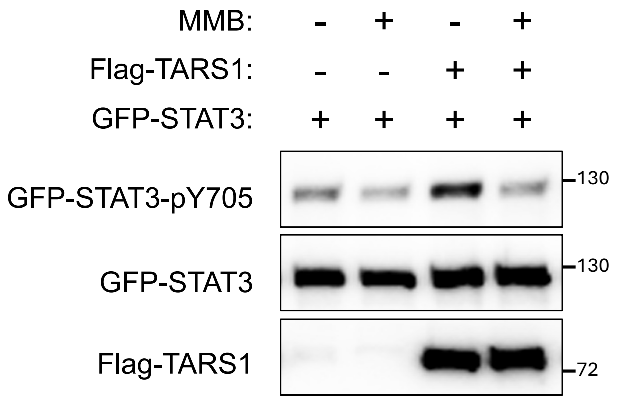


**Figure S7: TARS1 activation of STAT3 is dependent on JAK activity**
HEK293 cells were transfected with GFP-STAT3 with or without Flag-TARS1 for 48 hours, followed by treatment with 2.5 μM momelotinib (MMB) or DMSO for 60 mins. Western blots shown are representative of 3 independent experiments.
