# Supplementary material for: Threonyl-tRNA synthetase activates STAT3 by a nontranslational mechanism[image]
Source: J Biol Chem. 2025 Dec 9;302(2):111032. doi: 10.1016/j.jbc.2025.111032 (PMC12818216; doi:10.1016/j.jbc.2025.111032)
Supplement: Supporting Information [file mmc2.pdf]

SUPPORTING INFORMATION – MATERIALS AND METHODS

Antibodies

Antibodies for the following proteins were obtained from Cell Signaling Technology (Danvers, MA, USA): STAT3 (#12640), phospho-STAT3 (Y705; #9138), phospho-STAT3 (S727; #9134), JAK2 (#3230), phospho-JAK2 (#3776), ERK1/2 (#4695), phospho-ERK1/2 (Thr202/Tyr204; #4370), p38 MAPK (#9212), phospho-p38 MAPK (Thr180/Tyr182; #4511), AKT (#9272), phospho-AKT (Thr308; #9275), S6K1 (#34475), phospho-S6K1 (Thr389; #9243). Antibodies for TARS1 were obtained from Abcam (Cambridge, MA, USA. #ab236903) and Santa Cruz Biotechnology (#sc-166211). Anti-Flag antibodies were from Sigma-Aldrich (for immunofluorescence, #F9219) and Proteintech (for western blotting, #20543-1-AP). Anti-GFP (#66002-1-Ig) and anti-tubulin (#80762-1-RR) antibodies were from Proteintech.

Plasmids

pcDNA3-Flag-TARS1 (mouse cDNA) was previously reported [29], and was used to generate all point mutants of TARS1 using the Q5® Site-Directed Mutagenesis Kit (New England Biolabs, Ipswich, MA, USA) and truncation mutants using PCR. GFP-TARS1, GFP-DARS1, GFP-KARS1, and GFP-EPRS were created by subcloning human cDNAs into pcDNA3-EGFP, a gift from Doug Goldenbock (Addgene, plasmid #13031). GFP-LARS1 was purchased from Sino Biological (LARS1-GFP-Spark). All the constructs above have GFP at the C-terminus. Human STAT3 cDNA was a gift from the laboratory of James Darnell [43] and then subcloned into pcDNA3-Flag or pcDNA3-EGFP described above. The following plasmids were obtained from Addgene (Watertown, MA, USA): STAT3-C-Flag (#8722, [12]) and STAT3 luciferase reporter (4x M67 pTATA Tk-Luc) (#8688, [34]) were gifts from James Darnell; Flag-JAK1 (#174572, [44]) was a gift from Mathew Garnett; pCMV-dR8.2 dvpr (#8455, [45]) and pCMV-VSV-G (#8454, [45]) were gifts from Robert Weinberg.

Cell culture and transfection

All cell lines used in this study were originally obtained from ATCC and authenticated by the Tumor Engineering and Phenotyping Shared Resource at the Cancer Center of Illinois. Cells were monitored for mycoplasma contamination regularly, and all cells were mycoplasma-negative except for HEK293 cells used for the co-IP experiments. Transfection of H1703 cells was performed using Lipofectamine ™ 3000 (Invitrogen, # L3000008) in 12-well plates. Briefly, 1 µg of plasmid DNA was mixed with 2 µL of P3000 in 100 µL Opti-MEM™, then combined with 1 µL of Lipofectamine 3000. After 15-min incubation at room temperature, the mixture was added to the cells. Medium was replaced after 4 hrs and cells were collected 72 hrs post-transfection. For experiments in HEK293, cells were transfected in 12-well or 6-cm plates using polyethylenimine (PEI) at 3 µL/µg of DNA. The DNA:PEI mixture was incubated at room temperature for 15 min before adding to the cells.

Lentivirus-delivered RNA interference

shRNA in the pLKO.1-puro vector targeting human TARS1 was previously reported [29] and obtained from Sigma-Aldrich (clone ID: TRCN0000045680). A hairpin of scrambled sequence (shScramble) used for a negative control and lentivirus packaging plasmids were previously described [45]. For lentiviral packaging, pLKO-shRNA DNA, pCMV-dR8.2 dvpr, and pCMV-VSV-G were co-transfected at 4 µg, 3.6 µg, and 0.4 µg into HEK293T cells using polyethyleneimine (PEI) (Avantor-VWR, AAA43896-01) in a 10-cm plate. The medium was changed 6-8 hrs post-transfection, and the medium containing viruses was collected 24–48 hrs post-transfection. NSCLC cell lines were transduced in growth medium with virus plus 8 µg/mL polybrene and selected in 2 µg/mL puromycin for 48–96 hrs, followed by re-seeding into 12-well plates.

Cell viability assay

Cells were trypsinized to harvest and resuspended in an appropriate volume of culture medium. An equal volume of 0.4% Trypan Blue solution (Thermo Fisher Scientific, #15250061) was mixed with the cell suspension and incubated for 2-3 min at room temperature. The mixture was then loaded onto a hemocytometer, and viable (unstained) cells were counted under a light microscope. Each sample was counted in duplicate to ensure accuracy.

Cell proliferation assay

To assess cell proliferation, EdU labeling was performed as previously described [46]. Briefly, after 96 hrs of selection in puromycin, H1703 cells were incubated with EdU (final concentration 1 µM) for 4 hrs. Upon fixation with 3.7% formaldehyde, cells were treated with PBS containing 10 µM FAM azide 5-isomer, 1 mM $CuSO4$, and 100 mM ascorbic acid for 30 min, followed by DAPI staining for 30 min. Cells were imaged with a Leica DMI 4000B fluorescence microscope (Leica, Wetzlar, Germany). The fluorescence images were captured using a RETIGA EXi camera (QImaging, Surry, BC, Canada) and Image Pro Express software (Media Cybernetics, Rockville, MD, USA). Images were analyzed using ImageJ (NIH, https://imagej-nih-gov.proxy2.library.illinois.edu/ij/).

TUNEL assay

To assess apoptosis, cells were fixed using 3.7% formaldehyde and TUNEL assays were performed using the Click-iT™ TUNEL Alexa Fluor Imaging Assay Kit (Thermo Fisher Scientific, #C10245) according to the manufacturer's instructions. Fluorescence microscopy and quantification were performed as described in "Cell proliferation assay" above.

Mouse xenograft

Female athymic nude mice were purchased from Charles River Laboratories and grafted with H1703 derivative cell lines into the flank. Assessment for palpable tumors occurred at least two times per week post-graft and resulting tumor sizes were measured with calipers as we have done previously [47-49]. Data of tumor-free survival was presented as time to first palpable tumor in a Kaplan-Meier survival curve and subjected to log-rank (Mantel-Cox) test for statistical analysis. Tumors were resected after reaching at least ~1000 mm^3 or when excessive scratching by the animal occurred. Resected tumors were fixed in formaldehyde, transferred to 70% ethanol, embedded in paraffin, and sectioned, followed by hematoxylin and eosin (H&E) staining for histological analysis.

Luciferase assay

H1703 cells grown in 12-well plates were transduced with either shScramble or shTARS1 lentivirus and selected for 48 hrs in 2 µg/ml puromycin. Cells of equal numbers were then plated on fresh plates in growth medium. Next day, cells were transfected with the STAT3 reporter plasmid for 48 hrs. For rescue experiments, empty vector, WT-TARS1 or mutants were co-transfected with the reporter plasmid. Cells were lysed with 1x passive lysis buffer (#E1910, Promega, Madison, WI, USA) under gentle shaking at room temperature for 15 min. The lysate was cleared in a microcentrifuge (17000 rpm; 30 sec), and the supernatant was subjected to assay using the Luciferase Reporter Assay System (Cat #E1500, Promega, Madison, WI, USA) on an Agilent BioTek Synergy LX Multimode plate reader.

Immunofluorescence staining

H1703 cells were seeded on a 12-well plate in growth medium after 48 hrs of puromycin selection upon TARS1 knockdown. Cells were transfected with either WT or mutant Flag-TARS1 for 72 hrs. After transfection cells were incubated with EdU (final concentration 1 µM) for 4 hrs. Samples were fixed using 3.7% formaldehyde. EdU signals were developed using the method

described above, followed by immunostaining with an anti-Flag antibody. Briefly, cells were permeabilized with 0.2% Triton X-100 for 10 min following a 30-min block with 5% bovine serum albumin (BSA). Samples were then incubated with an anti-Flag antibody (1:100) for 2 hrs at room temperature. After rinsing with PBS containing 0.05% tween-20 (PBST), secondary antibody (Alexa Fluor 594-conjugated anti-mouse IgG, 1:50) was applied for 1 hr. Samples were washed three times with PBST, counterstained with DAPI, and imaged. Fluorescence microscopy and image analysis were performed as described in the "Cell proliferation assay" section above. Flag-positive and Flag-negative cells on the same images were quantified separately for EdU signals to determine proliferation of transfected and non-transfected cells, respectively.

Cell lysis and western blotting

For western blotting, cells were rinsed with PBS and lysed in SDS sample buffer containing 5% β-mercaptoethanol. Proteins were resolved on SDS-PAGE, transferred onto PVDF membrane (EMD Millipore, Darmstadt, Germany), and incubated with various antibodies according to the manufacturers' recommendations. Detection of horseradish peroxidase-conjugated secondary antibodies was performed with SuperSignal West Pico PLUS Chemiluminescent Substrate (Thermo Fisher Scientific, Waltham, MA, USA) and visualized using an iBright CL1000 or CL1500 Imaging System (Thermo Fisher Scientific). Quantification of western results were performed by densitometry in ImageJ (NIH, https://imagej-nih-gov.proxy2.library.illinois.edu/ij/). Equal loading of protein samples within an experiment on a protein gel was confirmed by blotting for tubulin as a house-keeping protein in lysates. However, we note that tubulin has not been validated to be unaffected by all experimental conditions in this study. Tubulin levels were not used for quantification of data except for Fig. 1A and Fig. S2A. The activity of each signaling protein was expressed as the ratio of phosphorylation level to total level of that protein, independent of the tubulin level.

Immunoprecipitation

HEK293 cells were seeded in 6-cm dishes at 60-80% confluency, transfected for 48 hrs, followed by lysis in 20 mM Tris (pH 7.5), 2 mM EDTA, 2 mM EGTA, 0.1 mM Na_3VO_4, 25 mM NaF, 25 mM β-glycerophosphate, 150 mM NaCl, 1% NP-40, and 1x Protease inhibitor cocktail (Sigma-Aldrich; Cat #P8340). The cell lysates were cleared with a microcentrifuge at 13,000 rpm for 7 min at 4 °C. Flag-tagged proteins were immunoprecipitated from the lysates by 20-min incubation with anti-FLAG M2 Affinity Gel (Sigma-Aldrich; #A2220) followed by three washes with 500 µL lysis buffer each. The beads were resuspended in SDS sample buffer and heated at 95 °C for 5 min, followed by SDS-PAGE and western blotting. For immunoprecipitation of endogenous proteins, H1703 cells were seeded in 6-cm dishes at 85% confluency, followed by lysis in the aforementioned buffer and cleared by centrifugation. The cleared lysate was incubated with antibodies at 4 °C overnight, followed by incubation with Protein A agarose at 4 °C for 2 hrs. The beads were washed three times with the lysis buffer and then boiled in SDS sample buffer.

Aminoacylation assay

HEK293 cells were transfected with Flag-TARS1 (WT or mutants) or empty vector for 24 hrs. Cell lysates were subjected to anti-Flag immunoprecipitation as described above. The first wash was done in the cell lysis buffer, and the second wash was in 25 mM Tris-HCl pH 7.4, 150 mM NaCl, 1% NP-40, and 5% glycerol. tRNA aminoacylation assay was performed following a published protocol [50] with some modifications. To the immunocomplex on beads, 20 µL assay buffer was added containing 20 mM HEPES (pH 8.0), 100 mM NaCl, 5mM $MgCl_2$, 3mM ATP, 1mM DTT, and 20 µg/µL yeast tRNA. The reaction mixture was equilibrated to 30 °C in a thermomixer (Eppendorf), and the reaction was initiated by the addition of ^{3}H-threonine (with unlabeled threonine) to a final concentration of 150 µM. Following incubation at 30 °C for 30 min under gentle agitation, 10-µL aliquots were spotted on filter pads (Whatman®; #1003323) pre-soaked

with 10% trichloroacetic acid (TCA) and 0.5% Casamino acids. The filters were washed three times for 10 mins each with cold 5% TCA solution, followed by a brief rinse once with cold 70% ethanol and once with anhydrous ether. The washed pads were then dried under a heat lamp. Radioactivity was determined by liquid scintillation counting (Beckman Coulter LS 6500). Activity of the recombinant protein was calculated by subtracting the background (empty vector sample) and normalizing to WT-TRAS1 as 100%.
